# Blood Pressure and Penumbral Sustenance in Stroke from Large Vessel Occlusion

**DOI:** 10.3389/fneur.2017.00317

**Published:** 2017-07-03

**Authors:** Robert W. Regenhardt, Alvin S. Das, Christopher J. Stapleton, Ronil V. Chandra, James D. Rabinov, Aman B. Patel, Joshua A. Hirsch, Thabele M. Leslie-Mazwi

**Affiliations:** ^1^Department of Neurology, Massachusetts General Hospital, Harvard Medical School, Boston, MA, United States; ^2^Neuroendovascular Service, Massachusetts General Hospital, Harvard Medical School, Boston, MA, United States; ^3^Department of Neurosurgery, Massachusetts General Hospital, Harvard Medical School, Boston, MA, United States; ^4^Interventional Neuroradiology, Monash Imaging, Monash Health, Monash University, Melbourne, VIC, Australia

**Keywords:** stroke, penumbra, blood pressure, thrombectomy, pressor therapy, neuroprotection

## Abstract

The global burden of stroke remains high, and of the various subtypes of stroke, large vessel occlusions (LVOs) account for the largest proportion of stroke-related death and disability. Several randomized controlled trials in 2015 changed the landscape of stroke care worldwide, with endovascular thrombectomy (ET) now the standard of care for all eligible patients. With the proven success of this therapy, there is a renewed focus on penumbral sustenance. In this review, we describe the ischemic penumbra, collateral circulation, autoregulation, and imaging assessment of the penumbra. Blood pressure goals in acute stroke remain controversial, and we review the current data and suggest an approach for induced hypertension in the acute treatment of patients with LVOs. Finally, in addition to reperfusion and enhanced perfusion, efforts focused on developing therapeutic targets that afford neuroprotection and augment neural repair will gain increasing importance. ET has revolutionized stroke care, and future emphasis will be placed on promoting penumbral sustenance, which will increase patient eligibility for this highly effective therapy and reduce overall stroke-related death and disability.

## The Evolving Landscape of Stroke Care

Stroke is the second leading cause of death in the world and the leading cause of disability in the United States (1, 2). Strokes can broadly be classified as either ischemic (approximately 80–90%) or hemorrhagic (approximately 10–20%) (3). Among ischemic strokes, there exist two major etiologies: large vessel occlusions (LVOs) and small vessel disease (SVD). LVO can occur from embolization from a proximal source or less commonly *in situ* atherosclerosis. SVD, on the other hand, may result from several processes including lipohyalinosis (4). Approximately one-third of ischemic strokes are LVO (5), which account for the majority of stroke-associated mortality and morbidity (6).

The treatment of stroke, and particularly LVO, focuses on restoration of blood flow to the ischemic penumbral tissue. This is accomplished by eliminating the primary site of obstruction resulting in reperfusion (7, 8). Since the NINDS recombinant tissue plasminogen activator (tPA) trial in 1995 (9), intravenous tPA has been the mainstay of stroke therapy. Six additional trials, including ECASS (10), ECASS II (11), ATLANTIS A/B (12, 13), ECASS III (14), EPITHET (15, 16), International Stroke Trial (IST)-3 (17, 18) further cemented this therapy. Based on results from these trials, for all acute ischemic strokes evaluated within the 4.5 h window meeting certain criteria, the current standard of care is treatment with intravenous tPA (dosed at 0.9 mg/kg with 10% given as a bolus and the remainder over a 60-min infusion). However, there is some controversy regarding its efficacy for LVO, in which the thrombus burden is often large. Recanalization may be as low as 8% of LVO patients treated with thrombolytic therapy alone (19), and outcome is often poor despite IV tPA.

In 2015, there was a paradigm shift in the treatment of LVO with the demonstration of efficacy and safety of endovascular thrombectomy (ET) (20–24). This represented the culmination of many years of trials refining the understanding and establishing the benefit of endovascular stroke therapy, starting with the PROACT-II trials, where intra-arterial urokinase increased rates of recanalization compared to heparin alone (25, 26). The outcomes of patients with LVO was revolutionized after the recent ET trials, which included magnetic resonance (MR) CLEAN, REVASCAT, ESCAPE, SWIFT PRIME, EXTEND IA, and more recently THRACE (20–24, 27). All successfully demonstrated a significant benefit over IV tPA alone.

This new and highly effective approach to the treatment of patients with LVO has energized a thorough assessment of the delivery of ET to patients who are eligible. How can we optimize outcomes for this patient population? In treating LVO, several approaches should be considered, including

(1) rapidly eliminating the primary site of obstruction to restore blood flow and reperfuse ischemic tissue,
(2) aiding in collateral perfusion to augment blood flow to the ischemic penumbra, and/or
(3) introducing agents that could sustain penumbral tissue and aid repair of the neurovascular unit.

## The Ischemic Penumbra and a Renewed Focus on Penumbral Sustenance

The concept of the “ischemic penumbra” was first described by Astrup and colleagues as an area of brain surrounding infarcted tissue where electrical failure was present, but ion pump failure had not yet occurred (7). This area is a viable target for therapeutic intervention as tissue has the potential to be restored to baseline function. An important goal of stroke therapy is improving blood flow to ischemic tissue. This has been the prominent focus of efforts directed to care in the current LVO era. However, as there are frequent delays in triaging patients from the site of initial evaluation to a comprehensive stroke center capable of ET, there is great interest in devising interventions to increase “penumbral sustenance” and sustain or save viable tissue until reperfusion can occur.

Blood pressure (BP) management is part of an encompassing strategy with this goal in mind. The concept of approaches directed to BP manipulation is to aid the collateral perfusion by allowing permissive hypertension or in some cases inducing hypertension. This supports or restores blood flow to ischemic penumbral tissue. Beyond restoring blood flow, another theoretical target is to protect the penumbral tissue at risk of death and/or enhance its repair. The target tissue is a complex “neurovascular unit,” which has been well described for LVO (28–30). It encompasses the interactions between neurons, astrocytes, microglia, endothelial cells, and smooth muscle cells. Together, the unit is involved in maintenance of synapses, regulation of neurotransmitters, energy metabolism, blood–brain barrier, and blood flow.

**Figure 1:**
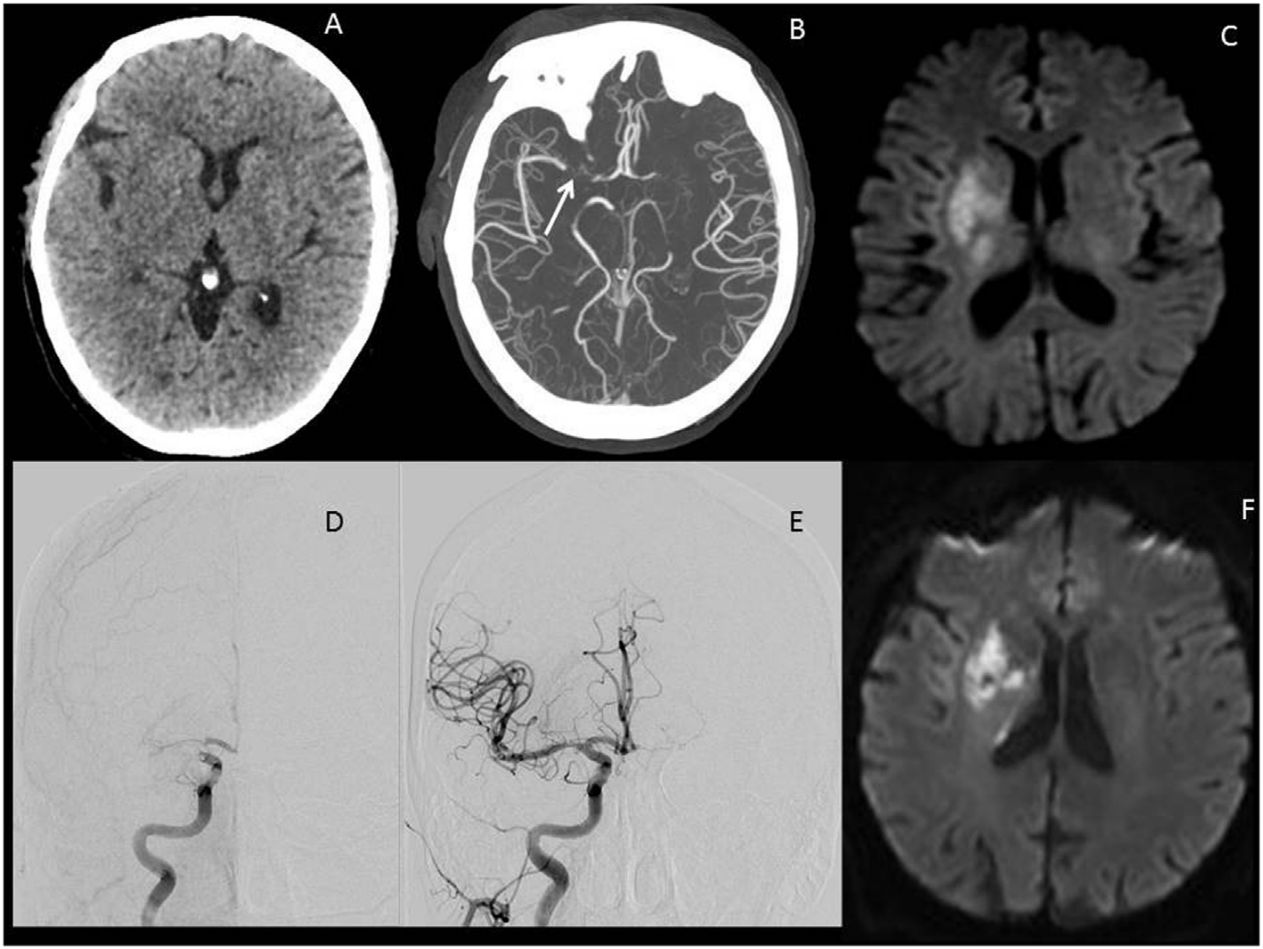
Illustrative images of a large vessel occlusion (LVO) stroke patient. Patient was a 67-year-old male presenting 4 h after onset with a full right middle cerebral artery (MCA) syndrome due to right MCA occlusion, NIHSS 14. **(A)** Emergent head computerized tomography without hemorrhage as a cause of stroke syndrome. **(B)** Axial maximal intensity projections from CTA showing right MCA occlusion (white arrow). **(C)** Emergent MRI DWI showing a small established core infarct. On the basis of this combined imaging and clinical data, it was determined that the patient had a large penumbra and small region of established injury and was therefore a good candidate for reperfusion therapy. **(D)** Anteroposterior view, catheter angiogram. The right internal carotid artery (ICA) injection reveals thrombus at the carotid terminus with only minimal anterior cerebral artery (ACA) opacification seen. Findings are consistent with an ICA-T occlusion. **(E)** Complete recanalization following mechanical thrombectomy, with full reperfusion (not shown) of the threatened penumbra. **(F)** 24 h MRI DWI showing arrest of infarct growth following reperfusion of the penumbra. The patient improved to NIHSS 4 by discharge on day 3 post-op. His stroke was determined to be cardioembolic following detection of atrial fibrillation after complete evaluation for cause, and he was free of deficits at 90-day follow-up.

The concept of the ischemic penumbra has guided acute stroke therapy for several decades now. In the current era of effective reperfusion, assessing core and penumbral tissue has become an important component of selection for therapy. All LVO patients have infarcted core and ischemic penumbral tissue (31). In the context of LVO, core and penumbra are often inversely related: a small core implies a large penumbra. Reperfusion keeps the core volume small, or prevents it from growing, an essential consideration given that core is a powerful biomarker for patient outcome (32). The best LVO candidates for ET have small ischemic cores, significant clinical deficits (large penumbra at risk), and an LVO amenable to thrombectomy. Reperfusion by thrombectomy preserves the penumbra and limits growth of the ischemic core. Figure 1 illustrates imaging findings for one such patient.

While the safety of ET in patients with large ischemic cores [Diffusion Weighted Imaging (DWI) lesion >70 cm^3^ or Alberta Stroke Program Early CT Score (ASPECTS) < 5] is not well established, ET is typically considered to have limited benefit as the infarct may be “completed” (33, 34). However, this concept has recently been challenged with one in three patients with pre-treatment DWI volumes >70 cm^3^ and successful reperfusion achieving good functional outcome (35, 36). The time window from last seen well to intervention has been perhaps one of the more controversial inclusion criteria. While time is a proxy for viable penumbra, and viable penumbra decreases as a function of time, there is great variability from patient to patient. In a cohort of patients with LVO, over 75% maintained perfusion–diffusion mismatch (described subsequently) at 9 h after symptom onset (37) and a small portion even at 24 h (38). This appears to be most related to the adequacy of collaterals and was used as the basis of selection in the ESCAPE trial (22). Conversely, a “malignant” collateral profile is highly specific for a large core infarct (39). The collateral circulation is therefore a patient specific variable in the approach to BP modulation, and in a broader sense penumbral salvage, in LVO patients.

## Collateral Circulation

Cerebral blood flow (CBF) is impaired in LVO, as the vascular territory affected contains an area of severely reduced blood flow (the core), and moderately reduced blood flow (the penumbra). Below a CBF of 10 ml/100 g of tissue/min, infarction of brain tissue occurs; however, between 10 and 20 ml/100 g of tissue/min, the brain can withstand several hours of ischemia leaving the penumbra viable (7, 40). Perfusion to the penumbra is accomplished by collateral blood flow through the collateral circulation. This circulation consists of vessels that compensate for reductions in CBF to the penumbra when large vessels are occluded. Therefore, a goal of stroke therapy should be to augment collateralization to preserve the penumbra, reducing the area of infarction, either in lieu of or while awaiting definitive reperfusion.

**Figure 2:**
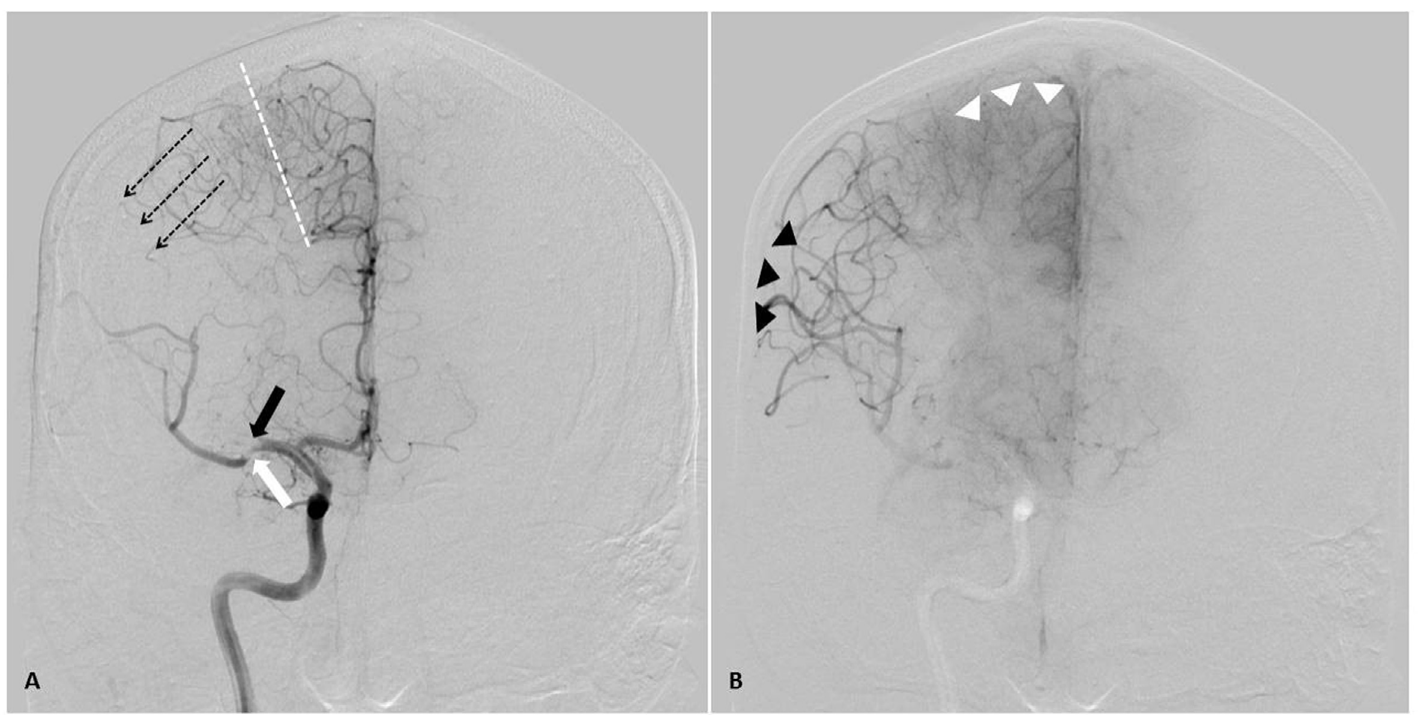
AP angiographic images demonstrating adaptations of poorly perfused cerebral tissue, right internal carotid injection. Right middle cerebral artery (MCA) stroke, demonstrating poorly perfused but viable penumbral tissue as a therapeutic target through elevation of blood pressure. **(A)** Mid arterial phase. Black arrow: complete occlusion of MCA superior division. White arrow: partial occlusion of MCA inferior division. Dotted line: watershed zone between anterior and middle cerebral arteries. Small caliber vessels in this region represent leptomeningeal collaterals. Note early retrograde filling of distal superior division MCA territory (dotted arrow). **(B)** Late arterial phase. Black arrow heads demonstrate delayed antegrade fill of the inferior division and delayed retrograde fill of the superior division MCA branches. Note the maximal dilatation of these vessels, and the delay to parenchymal opacification when compared to the normally perfused anterior cerebral artery territory (white arrow heads). Both the partial occlusion with antegrade flow and the complete occlusion with retrograde collateral support benefit from systemic hypertensive responses, given the pressure passivity of the dilated distal MCA vasculature.

The collateral circulation is a complex network of vessels that is comprised of various conduits between the internal carotid artery (ICA), the external carotid artery, branches of the ICA, and the posterior cerebral circulation (PCA). Flow occurs down pressure gradients, as in all vascular beds. After vascular occlusion, the abrupt change in gradients allows flow to be redirected rapidly from perfused vascular beds into the ischemic tissue. Flow augmentation through collaterals occurs *via* one of two ways. First, flow will increase through primary collaterals, which are existing anastomoses (such as the vessels of the Circle of Willis) to supply ischemic tissue. The second method is through secondary collaterals (such as pial–pial leptomeningeal collaterals), which may or may not be anatomically present. These collaterals develop and increase as a response to increased oxygen demand in the brain (41). Figure 2 exemplifies adaptations of poorly perfused cerebral tissue, including the role of collateral circulation.

## Cerebral Autoregulation and BP

To better understand how BP can contribute to normal and abnormal cerebral brain function, it can be considered a proxy for several physiologic processes. The brain maintains a high level of metabolism that requires tight coupling of energy supply and demand. First described in an animal model in the 1930s, the term autoregulation was coined twenty years later by Lassen and marked the acceptance of this phenomenon as central to brain physiology (42). Despite similarities to other end organs, the brain’s microcirculation has unique anatomy and physiology. CBF (*Q*) is directly related to pressure (*P*) and inversely related to resistance (*R*), described mathematically by *Q* = *P*/*R* (43). By reflexively modifying resistance, the cerebral vasculature can adjust to acute changes to maintain homeostasis in metabolic supply and demand. Through autoregulation, the cerebral vasculature maintains a relatively constant CBF between perfusion pressures of 50 and 150 mm Hg. Resistance is modified through pressure dependent actions, sensed by changes in wall tension, on smooth muscle cells of the precapillary arterioles. This process has been termed myogenic autoregulation (44).

In addition to these mechanical effects of perfusion pressure, the cerebral vasculature can also modify resistance in response to the local metabolic and chemical milieu. Local flow undergoes change with local brain function by as much as 10–20% in response to mediators such as the hydrogen ion, carbon dioxide, potassium, adenosine, glycolytic intermediates, phospholipid metabolites, and nitric oxide. Compared to the microvasculature of other organs, the cerebral vasculature has decreased dependence on sympathetic and parasympathetic innervation; maximal stimulation of either results in only minor changes in CBF. Furthermore, this neurovascular coupling is primarily at the level of the large arterioles and small arteries which are innervated in their adventitia (45). Interestingly, the posterior circulation has less sympathetic innervation and may be more susceptible to injury secondary to high perfusion pressures (as seen in posterior reversible encephalopathy syndrome and pre-eclampsia, for example).

**Figure 3:**
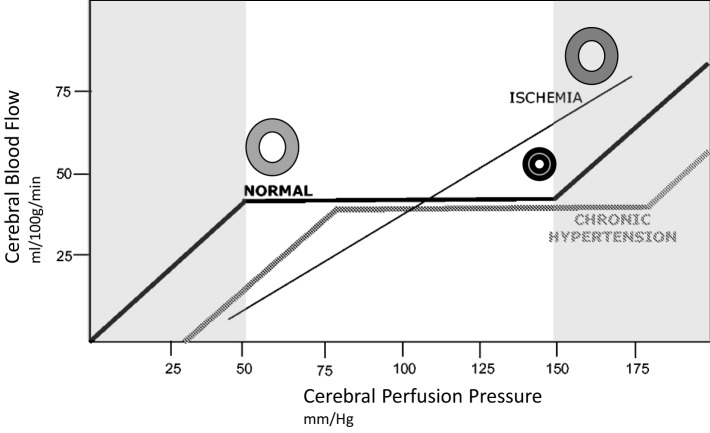
Idealized relationship between cerebral perfusion pressure (CPP) and cerebral blood flow (CBF). The normal autoregulation curve maintains a constant CBF over a range of CPP. At the lower end of the autoregulation range, vessels are dilated to encourage flow, at the upper end of the range they are maximally constricted, represented by circles in the illustration. Chronic hypertension moves the entire curve to the right. In ischemic brain, vasculature is maximally dilated, and the ability to autoregulate is lost. This introduces pressure passivity, where changes in CPP (through blood pressure modulation) are directly transmitted to CBF. The CBF is therefore passively dependent on the mean arterial pressure.

Cerebral autoregulation is dramatically impaired after stroke. This occurs because of maximal dilatation of arterioles in the context of ischemia (46). The consequence of this dilatation is that CBF becomes passively dependent on mean arterial pressure (MAP) (47). In the presence of ischemia, cerebral vessels fail to alter their vasoconstrictive response (i.e., inability to adjust resistance) in response to changing perfusion pressure (48). By increasing systemic BP, a corresponding increase in perfusion to the ischemic and hypoperfused brain occurs. Figure 3 illustrates this relationship for normal, chronically hypertensive, and ischemic cerebral tissue. Pathophysiologically, we consider BP, a ready variable to measure, because it represents a proxy for CBF (higher pressures, higher flow), given evidence of direct correlation between these two parameters in patients experiencing ischemia. CBF, as approximated by mean flow velocity, can be increased with elevation of MAP (49), or decreased with depression of MAP (50). Blood flow in turn is a proxy for delivery of oxygen and glucose, and removal of waste products, which allows for preserved or restored neuronal function.

## Assessing for Penumbral Tissue in LVO Patients

Evaluation of the penumbra through clinical or imaging techniques is essential in the effort to better modify the outcomes of LVO stroke patients. Different methods of assessing penumbra have been pursued, with ongoing controversy about optimal thresholds and techniques. As stated, determining how much viable ischemic tissue remains in relation to how much has already become infarcted core is valuable in decision making. While there are various algorithms for assessing areas of decreased flow with perfusion weighted imaging (PWI), many now believe that a clinical deficit out of proportion to the ischemic core on DWI is reliable for treatment decisions. These differences are referred to as perfusion–diffusion mismatch and clinical–diffusion mismatch, respectively. The presence of penumbra can also be established entirely clinically in select patients with a BP- or position-dependent exam. Clearly, there is penumbra that is viable if its function is restored by flattening the head position or raising the BP. Interestingly, a review of the Harvard Stroke Registry showed that 20% of all strokes progressed after onset, and of those, 33% were secondary to LVO (51, 52).

While its use is controversial, PWI may aid in identifying the volume of tissue at risk (31, 53). Both computerized tomography (CT) and MR PWI rely on whole brain imaging during the passage of an intravascular contrast bolus through tissue capillaries. This dynamic information is used to render 3D cross-sectional images that represent capillary blood flow (54). These changes in contrast in each voxel can be used to calculate several parameters according to the central volume principle (55). A time-density curve is made so that change in contrast is plotted against time in seconds. The area under this curve is the cerebral blood volume (CBV) (56, 57). CBV represents an estimate of the total volume of blood in the intravascular space per voxel (mL blood/100 g tissue). CBV is dependent on the microvasculature and is dramatically decreased in areas of lost perfusion, such as infarcted core (58). The mean transit time (MTT) is the average time in seconds between arrival of contrast to a given voxel and the outflow from that voxel into the venous system (59). MTT is dependent on microvasculature anatomy (capillary occlusion and tortuosity of collaterals) and on local perfusion pressure (60). An area of prolonged MTT indicates an area that is hypoperfused, and this hypoperfused region that extends beyond the core may be used to represent the penumbra. CBF is the volume of blood flowing through a given voxel per time (mL blood/100 g tissue/min). The central volume principle states that CBF is the ratio of CBV to MTT. One additional parameter that can be helpful is the time to bolus peak (TTP). TTP is an estimate of the time (seconds) between arrival of contrast at the precapillary arteriole and peak concentration of contrast in the capillary bed.

In acute stroke, decreased cerebral perfusion pressure (CPP) triggers small arterial and arteriolar dilation by autoregulation, as previously described. This results in a compensatory increase in CBV and a prolongation of MTT and TTP to allow for maintenance of CBF. However, when CPP drops so low that autoregulation fails, CBF is decreased and ischemia or infarction occurs (61). Clinically, CBV, CBF, and MTT are encoded in color maps to aid in PWI interpretation. In the evaluation of acute stroke, CT has the advantages of speed, low cost, and wide-spread availability (31, 54). MR, on the other hand, has the advantage of DWI, which is the gold standard for predicting ischemic core (62). It should be noted that the clinical benefit of using PWI remains unknown, and thus the AHA/ASA guidelines do not provide any recommendation that PWI be used in the acute setting (63).

Nonetheless, increasing attention has been paid to the use and automation of perfusion imaging. Several recent trials, including SWIFT Prime (24), EXTEND-IA (21), DAWN (64), and DEFUSE 3 (65) utilized RAPID software (iSchemaView, California). While this is currently the most validated software, it is only one of several offerings on the market. These programs provide output in graphical and numerical form using the principles described above, with the goal of simplifying treatment decisions for stroke selection. Automation can be applied to measure core or penumbra and is becoming progressively more reliable, though still troubled by variable algorithms, intermittent inconsistency, and high implementation costs, all of which limit widespread utilization. These techniques are likely to play an increasing role in future stroke treatment decisions, particularly in marginal cases or later treatment windows, where penumbral sustenance gains increasing importance.

## Controversy about BP Targets in Stroke

Blood pressure modulation has long been considered for a potential role in improving survival of the penumbra. However, given the complexity and heterogeneity of ischemic stroke, well-defined BP targets are difficult to recommend and are often controversial. Indeed, several clinical trials examining various BP goals have yielded conflicting results. While primary and secondary prevention goals are more established, appropriate BP targets in acute stroke are less well described. The Eighth Report of the Joint National Committee on Prevention, Detection, Evaluation, and Treatment of High BP (JNC 8) provides excellent guidelines on BP management in the absence of stroke. It recommends a target BP of <150/90 for uncomplicated hypertensive patients in adults older than 60 years and <140/90 in adults less than 60 years, or patients with concurrent diabetes mellitus or chronic kidney disease (66). However, while BP in acute stroke has been examined extensively, there is limited or inconsistent data suggesting that BP modulation improves patient outcomes (67, 68).

Despite the variegated evidence regarding BP management in acute stroke, admission BP values do appear to carry prognostic information. It appears that both reduced and elevated BP are important, as demonstrated by a retrospective analysis of over 17,000 patients in the IST (69). In this study, the 2-week death rate from stroke increased by 17.9% for every 10 mm Hg admission systolic BP (SBP) below 150, and increased 3.8% for every 10 mm Hg above 150 (69). Furthermore, recurrent ischemic stroke within 2 weeks increased by 4% for every 10 mm Hg increase in SBP. This U-shaped curve has been reported in other retrospective and prospective studies (70, 71). However, a recent study suggests this association may depend on the revascularization status (72). To better understand these data from the IST trial, the controlling hypertension and hypotension immediately post stroke (CHHIPS) study randomized 180 patients within 36 h of symptom onset to receive pressor or depressor therapy to target a SBP goal of 150 (73). The study failed to recruit enough patients to the pressor arm (which had a goal of starting phenylephrine vs. placebo for SBP < 140 mm Hg) but did show that treating patients with SBP > 160 with labetalol or lisinopril had decreased mortality at 3 months compared to placebo. Furthermore, although the SBP was lower in the treatment group at 24 h, there was no increase in adverse events.

The results from the CHHIPS study is in accordance with other studies such as the Acute Candesartan Cilexetil Therapy in Stroke Survivors (ACCESS) study, a prospective, randomized, double-blind, placebo-controlled study which used candesartan cilexetil for early BP reduction in stroke (74). This study aimed for a BP reduction of 10–15% within 24 h of an acute stroke. After 12 months, there was a reduction in mortality and other vascular events, although there was no difference in functional dependency or cerebrovascular events. Interestingly, a more recent larger randomized, double-blinded, placebo-controlled study examined the angiotensin-receptor blocker candesartan for treatment of acute stroke (SCAST). This study showed no difference in outcomes in patients receiving candesartan for SBP > 140 mm Hg within 30 h of symptoms onset (75).

Other earlier studies, however, showed worse outcomes after BP reduction in acute stroke. One of the earliest trials, the low dose beta blockade in acute stroke (BEST) trial demonstrated an increase in mortality in patient receiving beta-blocker therapy after acute stroke (although there may have been selection bias among treatment groups). Another early randomized, double-blind, placebo-controlled trial, the INWEST trial, demonstrated worse outcomes following administration of nimodipine in the acute phase of stroke (76, 77). To add to the variation in results, studies such as the Continue or Stop Post-Stroke Antihypertensives Collaborative Study (COSSACS) demonstrated neutral outcomes upon lower BP on admission (by continuing home antihypertensive regimens) (78). A more recent study, the China Antihypertensive Trial in Acute Ischemic Stroke sought to lower SBP by 10–25% within the first 24 h after stroke (79). At 2 weeks, there was no differences in mortality or major disability in patients whose BP was reduced.

Collating the above data, two large Cochrane meta-analyses concluded that there is insufficient evidence to evaluate the effect of BP modulation on outcome after acute ischemic stroke (80, 81). However, many of these studies did not include subgroup analyses of stroke subtypes, specifically assessing for optimum BP targets in LVOs. This question is of critical concern given that many of these patients are candidates for ET, and peri-procedural BP management may have a very different consequence in this population dependent on collateral blood flow.

In the most recent guidelines of the American Heart Association (AHA) for the early management of patients with acute ischemic stroke, specific BP targets are not well defined but are dependent on several patient-specific factors (82). The guidelines do suggest stratification of BP targets in patients that receive intravenous thrombolytic therapy. In patients that are candidates for IV tPA, it is suggested that the BP be lowered to 185/110 prior to administration of therapy. From the time of tPA induction, BP monitoring is critical and should be checked every 15 min for 2 h, then every 30 min for 6 h, followed by every 60 min for 16 h. Once thrombolytic therapy has been introduced, the BP should be lowered further to 180/105, a target that should be maintained for 24 h with nicardipine or labetalol used as first-line agents. In patients with uncontrollable BP or diastolic BP > 140, sodium nitroprusside is suggested. In patients who are not candidates for IV tPA, the AHA guidelines advise against reducing BP unless it exceeds 220/120, unless there is an additional comorbid condition (such as myocardial infarction, congestive heart failure, or aortic dissection) that would benefit from BP reduction. In such cases, the guidelines suggest lowering the SBP by 15% and monitoring for any compromise of symptoms. These parameters apply to LVO patients being taken to the angiography suite for ET, which may occur in conjunction with or independent of IV tPA.

The authors of the 2013 AHA Acute Ischemic Stroke guidelines (an update is expected in late 2017 or early 2018) suggest temporary discontinuation of home antihypertensive regimens during the acute phase of stroke, and re-initiating this therapy 24 h after stroke onset. Hypotension is an uncommon event at the onset of stroke, and usually indicates another concurrent medical condition. The guidelines acknowledge the poor outcomes of patients with hypotension; however, a clear definition of hypotension (in the setting of stroke) is lacking, and is likely patient-specific. Correction of hypotension is suggested, and vasopressor agents are recommended if other means are ineffective.

## BP in LVO Stroke

The heterogeneity of stroke is likely one factor explaining the varying results seen in trials addressing BP in acute stroke. There are distinct etiologies of ischemic stroke, and it is likely that unique BP targets are optimal for each stroke subtype, or that certain subtypes are more sensitive to variations of BP than others. This remains to be definitively proven, but has a sound pathophysiologic basis. One prominent and widely used classification of stroke subtypes comes from the Trial of Org 10172 in Acute Stroke Treatment (TOAST) (83, 84). This trial defines five major subtypes of stroke: large-artery atherosclerosis, cardioembolism, small-artery occlusion (lacune), stroke of other determined etiology, and stroke of undetermined etiology. These subtypes are determined based on neuroimaging, echocardiography, and other laboratory data. LVO stroke results from either large-artery atherosclerotic disease or cardioembolism. As discussed above, it is likely that BP plays a more significant role with this subtype than others as a result of the potential for more meaningful collateral support given the proximal nature of the occlusion (85).

For patients with LVO, higher SBP on presentation is associated with lower revascularization rates in patients treated with tPA (86) and ET (87). There is a further association of high SBP on presentation with decreased collateral flow on pretreatment angiography (43). While these elevated SBP values may represent an effect and not a cause (i.e., severe occlusions that are difficult to reperfuse with poor collaterals are likely to cause higher SBP), several theories have been entertained. It is hypothesized that opposing hydromechanical forces, defined by anterograde flow proximal to the occlusion versus retrograde leptomeningeal collateral flow, may create a trans-clot pressure gradient (87).

Blood pressure has been shown to spontaneously decrease following reperfusion after LVO stroke (88). Patients with adequate recanalization 12 h after thrombolysis had lower values that those with inadequate recanalization (SBP 130 vs. 140). Furthermore, SBP remained elevated longer when recanalization failed. Similar results were shown in patients treated with ET, where those patients that recanalized demonstrated a greater fall than those who did not; the decline was significantly different from hours 8 to 12 post-procedure (89). This finding supports the construct that some of the marked elevation in presentation BP is the result of attempts to perfuse the ischemic brain through collateral circulation. The exact humoral or neural mechanisms by which local cerebral ischemia produces a systemic response remain unclear.

Based on these observational trends, it seems likely that in the patient with LVO, there are different BP goals depending on the setting (pre-ET, early post-ET, subacutely, and chronically). Prior to ET, increased BP is important for maintenance of perfusion through collaterals. Per the aforementioned guidelines, a reasonable systolic goal is <220mm Hg in the absence of tPA and <180 mm Hg after tPA administration (82). After recanalization *via* ET, it stands to reason that reduced BP is preferred, given that perfusion to previously ischemic tissue is restored. In this early post-ET period, it is reasonable to allow SBP autoregulation to <180 mm Hg and DBP to <105 mm Hg (43). Because there are no studies examining BP targets post-ET, the autoregulation goal of 180/105 has been extrapolated from guidelines for IV tPA (82, 90). Individual and institutional practices may vary, as some clinicians argue for lower targets such SBP < 160 mm Hg or even < 140 mm Hg to minimize reperfusion injury. However, BP targets post-ET should be patient-specific, evaluating the risk for both infarct extension, and hemorrhagic conversion. Factors that should be taken into consideration include high NIH Stroke Scale (NIHSS), ASPECTS score, hyperglycemia, use of concurrent antiplatelet or anti-thrombotic therapy, timing of recanalization, thrombolysis in cerebral infarction (91) score following reperfusion, persisting occlusions, mass effect or edema caused by infarction, location of infarction, and age (92). Increased concerns for possible hemorrhagic conversion should prompt use of lower ideal BP targets. Finally, in the subacute and chronic settings, there is no consensus guideline for BP targets following LVO stroke, though it likely requires tight control in the context of management of all vascular risk factors. Furthermore, the choice of antihypertensive regimen is also unclear. This should be individualized to each patient (93).

## Hypertension in LVO Stroke

From a physiological perspective, an argument can be made for permissive hypertension, or more affirmatively, induced hypertension in acute LVO patients. We have established above that the penumbra is dependent on maintenance of CPP for the preservation of blood flow to the ischemic area (94). Because these areas are already maximally dilated with loss of autoregulation, a minimal reduction in perfusion pressure may be detrimental to this pressure passive system (48). By inducing (or allowing) hypertension, the aim is to enhance CBF, and therefore neuronal support in penumbral tissue. Elevated hydrostatic pressure aims to increase perfusion through collaterals (leptomeningeal or through the Circle of Willis) and possibly through a partially occluded vessel (fixed stenosis versus lodged embolus) (94–96).

Despite the sound pathophysiologic arguments, it has been difficult to define specific parameters for BP targets and to prove efficacy of this therapy in clinical trials. Existing data are challenged by small retrospective studies, potential selection bias, and variability in therapeutic agents, BP targets, and protocols for BP elevation. Furthermore, there are limited data studying the chronic effects of augmenting perfusion in the acute setting. Several animal studies exploring therapeutic induction of hypertension in acute stroke have shown varying results. One early study showed increased cerebral edema in experimental animals following hypertension for MCA stroke (97). However, similar trials in rats showed that phenylephrine-induced hypertension applied 2 h after MCA occlusion resulted in improvement of ischemia as well as edema (98, 99). These hypertensive trials in rats have employed both hemodilution therapy as well as vasopressor therapy (100) and have demonstrated safety and efficacy (95). Further hypertensive trials evaluating prolonged hypertension showed increased risks of vasogenic edema, although short periods of hypertension still appeared safe and effective (101). Such findings have been replicated in rabbits, demonstrating benefit with acute periods of hypertension induction, but less benefit with prolonged therapy (102). There is also similar efficacy in canines, as studies administering vasopressor therapy within 1 h after occlusion demonstrated benefit (103). Studies in monkeys, inducing a 20–40% increase in BP by phenylephrine, demonstrated improvement in 50% of animals, although cardiac side effects did occur in some (104).

Several human studies examined induced hypertension, either by volume expansion or pressor therapy in acute ischemic stroke. Denny-Brown was perhaps the first to recognize improved neurological function associated with elevated BP (105). Several studies followed which attempted to augment BP through various vasoactive agents (106–109).

Perhaps the most widely studied vasoactive agent in acute stroke (both human and animal models) is phenylephrine, an α1 receptor agonist that increases BP by peripheral vasoconstriction. Compared to other agents, it is less likely to cause tachyarrhythmias as it has limited β receptor affinity, and the paucity of α1 receptors in the central nervous system means that inducing vasoconstriction in the cerebral vasculature is not a concern. Many of the studies employing phenylephrine were conducted independently by Rordorf and Hillis. One early study administered phenylephrine within 24 h of acute ischemic stroke. In 10 of 30 patients, a clear BP dependency was established and examination findings improved with pressor therapy. This study demonstrated safety of pressor therapy, given that no increased mortality or morbidity was observed (110). In a second prospective case series evaluating 13 patients, SBP was increased by 20%. 54% of patients responded to pressor therapy, with an average NIHSS decrease of 3.4 points. This study again demonstrated an efficacy, most notably in patients with large vessel atherosclerosis (85). Importantly, no adverse events were seen, reinforcing the safety of early pressor therapy in ischemic stroke. Hillis et al. demonstrated an improvement in language function in patients treated with phenylephrine within 24 h of stroke (111, 112). A subsequent randomized, prospective study using 15 patients administered pressor therapy to patients <7 h from the onset of acute ischemic stroke. 20% of these patients demonstrated a mismatch on PWI and were given either phenylephrine or IV fluids to target a goal BP >140 mm Hg or symptom improvement. After 3 days, an improvement in NIHSS and cognition was observed (113). Another study using PWI to guide clinical decision making demonstrated a mismatch in 15 patients 7 days after the onset of symptoms. Eight of these patients received pressor therapy, and six of those demonstrated improved function which correlated to decreased hypoperfused areas on neuroimaging (114).

In addition to phenylephrine, norepinephrine (NE) has been studied as a means of augmenting flow to preserve penumbra. The first of these studies was conducted by Schwarz et al. using 19 patients with large hemispheric strokes. Increases of 10 mm Hg in MAP were achieved by NE administration (49). This resulted in improved CPP from 72.2 to 97 mm Hg with a concomitant statistically significant (but not clinically significant) increase in intracerebral pressure (11.6 to 11.8 mm Hg). In a retrospective trial employing NE enrolling 34 patients (8 of which received IV tPA), 27% of patients responded to pressor therapy with a NIHSS change of 2, although 1 patient experienced cardiac arrhythmia and 2 others experienced intracerebral hemorrhage (115). These results suggest that while NE is effective, it should not be used as a first line agent given its propensity for tachyarrhythmia due to β1 receptor agonism. Other vasoactive agents including epinephrine (116), dobutamine (117), dopamine (118), and DCLHb (a hemoglobin-based oxygen-carrying solution) (119, 120) have been studied to a lesser degree and have demonstrated varying results, though none convincing for potential clinical application (121).

While the use of pressor therapy in acute stroke is controversial, there does appear to be a role in carefully selected patient populations. There are analogous scenarios in other disease states. For example, the use of induced hypertension to augment CBF in stroke shares some of the physiological basis applied to induced hypertension in subarachnoid hemorrhage patient experiencing vasospasm (122). Improving perfusion to threatened neuronal tissue is the goal. In stroke, large, randomized, placebo-controlled, blinded studies will need to be conducted to further evaluate its efficacy; however, from the limited data available, induced hypertension for acute ischemic stroke does appear to be safe, in the acute and subacute period (123).

## Suggested Approach to Induced Hypertension

Patients that are likely to benefit from induced hypertension are those with expected delay to ET, persistent LVO following ET (unsuccessful or partially successful recanalization), or those who are not candidates for ET for other reasons. Of these patients, those with a BP- or position-dependent examination, a DWI/clinical mismatch, or a DWI/PWI mismatch are the most likely to benefit. As there are risks associated with induced hypertension, some providers select only those patients that demonstrate symptomatic changes with BP fluctuations. Exposure to intravenous tPA is not a contraindication but limits the upper extent of BP elevation. That stated, the decision to initiate induced hypertension should not be taken lightly in patients who have been treated with thrombolytic therapy. Studies have shown higher BP is associated with worse outcomes and symptomatic hemorrhage in these patients (72, 124). Patients with intracranial hemorrhage, congestive heart failure, active coronary ischemia (changes on electrocardiogram or troponin leak), or existing autoregulation to systolic BP > 200 are generally excluded (85, 113).

**Figure 4:**
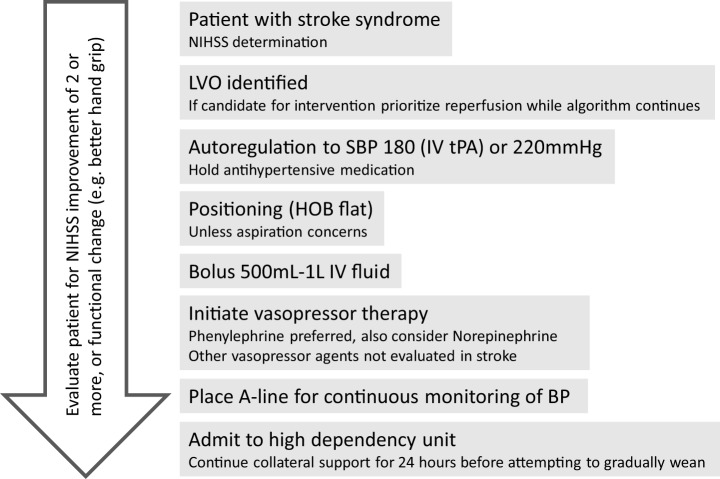
Algorithm for approach to hypertensive support for large vessel occlusion (LVO) patient. Reperfusion therapy should be the priority, but the algorithm provides for collateral support either while awaiting reperfusion (e.g., transfer to endovascular capable facility) or if reperfusion is not an option.

For the patient treated with therapeutic hypertension, we present an algorithm (Figure 4). A reasonable goal is to titrate to clinical response (i.e., improvement in neurologic exam) with a maximum SBP of 180 mm Hg if the patient has received IV tPA. Otherwise target specific goals, such as to increase MAP by 10–20% above baseline, should be pursued. This can be accomplished by holding antihypertensive medications, providing a fluid bolus, patient positioning with head of bed flat, and vasopressor therapy as indicated. Amongst pressors, phenylephrine should be the first line agent as it has been most studied and appears to be the safest; however, NE is a reasonable second-line alternative (85, 113). Any patient for which therapeutic induced hypertension is considered should be monitored in an intensive care setting with continuous arterial pressure monitoring and consideration of central venous access. To assess for side effects of induced hypertension, the following should be considered: chest X-ray, echocardiogram, serial electrocardiograms, central venous pressure, urine output, and standard daily laboratory evaluations (including frequent cycling of cardiac enzymes). Fortunately, the most deleterious side effects are also rare, including intracerebral hemorrhage, pulmonary edema, and cardiac arrhythmias (115, 123).

To support continuing induced hypertension, clinical or radiographic improvement should be observed. Different metrics have been used to assess clinical improvement, including the NIHSS, where improvements of four points or greater have been considered a positive response (85, 113). However, less demanding metrics could be considered, as improved fine motor ability or more fluent language output represent functionally significant improvement that would not be measured well with NIHSS. Duration of collateral support by induced hypertension should be sustained long enough to enable adaptation of these collateral connections. It is hypothesized that over 24 h is required for adaptation of collaterals, and that weaning pressors beyond 24 h is based on individual patient characteristics. Weaning should be accomplished gradually and with an immediate return to prior support levels if patient becomes symptomatic. Oral hypertensive agents (fludrocortisone, midodrine) could be utilized for patients who are unable to tolerate wean or for whom longer term support may be needed.

## Hypotension and Other Concerns in LVO Stroke

There are several important consequences of hypotension in stroke, especially with regard to LVO. When an LVO patient is hypotensive, it is first important to rule out additional pathology, such as myocardial infarction, dissection, or sepsis. That being stated, hypotension itself can lead to watershed infarctions (125) and is an important cause of transient ischemia attacks (126). Strokes caused by decreased perfusion can be secondary to low flow itself, or caused by an impaired clearance of microemboli in the setting of decreased CBF (127). Both of these scenarios are likely worsened by hypotension. Expectedly, if hypertension can augment perfusion, hypotension can worsen perfusion resulting in further ischemia. This is especially true in the setting of a fixed vascular lesion that may produce a low flow state. In the chronic setting, orthostatic hypotension has been shown to be a risk factor for ischemic stroke as seen in an analysis of the Atherosclerosis Risk in Communities (ARIC) study (128). A potential explanation for this finding is that transient decreases in BP may result in decreased cerebral perfusion *via* direct and indirect mechanisms (129). These findings have been extended to the acute setting in which both mean admission BP and discharge BP were shown to be predictors of mortality in stroke (130). As demonstrated by the U-shaped curve described previously (70), patients with admission mean SBP < 100 had an increased rate of death compared to those with higher BP. Likewise, patients with a discharge mean SBP < 120 had an increase in mortality compared to those with higher discharge BP (130). During the care of LVO patients, there are several instances where patients are at high risk of hypotension.

Volume status also plays an integral part in perfusion. During the acute phase of ischemic stroke, hypovolemia can cause cerebral hypoperfusion, as well as increase the risk of other conditions such as renal injury or myocardial infarction. The AHA/ASA recommends initiating maintenance isotonic fluids in euvolemic patients, as well as rapidly repleting intravascular volume in patients who are hypovolemic (82). Additional measures to manipulate volume status have been explored using volume expansion and hemodilution. Agents have been examined during acute stroke for their ability to modify blood viscosity (131) or expand effective circulating volume, through the use of crystalloid solutions (132) and albumin (133). These measures aim to increase CBF, yielding increased perfusion to the threatened penumbra. Indeed, animal models supported this idea (134), and several early studies in human acute stroke were promising (135, 136). However, in a Cochrane meta-analysis analyzing 3,100 patients in 18 trials, there was no mortality benefit or functional improvement with application of volume expansion regimens (131). They currently have no mainstream clinical role.

An intervention as simple as positioning the patient supine has been shown to allow increased venous return, cardiac output, and a 15–20% increase in CBF (137, 138). (More extreme positions can be considered, though there are only case reports supporting their experimentation. C. Miller Fisher, for example, was known to lift some patients by their feet with their heads near the floor to increase perfusion pressure (139)!) However, in patients at a high risk for aspiration, the risks and benefits of supine positioning should be weighed, with more evidence supporting the maintenance of the head of bead at a 30° angle (140). Patients with stroke symptoms may be positioned in the seated position by family members or ambulance crews, potentially worsening cerebral ischemia in the context of a proximal occlusion.

A further vulnerable period is when LVO patients are exposed to procedural sedation or general anesthesia during ET for LVO. Data currently are conflicted regarding the benefit of conscious sedation over general anesthesia. Limited randomized trial data are now available (141) with more data expected soon (142). There are data suggesting worse clinical outcomes for patients treated with general anesthesia (143, 144). Both induction and recovery phases of anesthesia are often associated with significant hemodynamic changes, which could result in collateral loss in the tenuous vessels perfusing the threatened penumbra. Hypotension and rapid BP fluctuations could exacerbate ischemic injury. Peri-procedural hypotension has been reported in endovascular stroke patients (145–147). Comparing procedural options, general anesthesia likely results in greater decreases in CPP than conscious sedation.

Anesthetic agents have varying effects on autoregulation of the cerebral vasculature, which may influence the ability to reach a therapeutic BP target. Therefore, the avoidance of anesthetic agents is generally preferred (43). Patients that do receive anesthesia should be monitored closely with frequent neurological examinations. With the exception of sevofluorane, most inhaled anesthetics significantly decrease CBF. For this reason, sevoflurane is a preferred inhaled agent for ET (43). Among the intravenous agents, propofol is the preferred agent because it does not affect cerebral autoregulation. For patients treated with conscious sedation Dexmedetomidine offers multiple advantages (148), with the caveat that bradycardia and hypotension can occur (149) and should be specifically anticipated and avoided.

During sleep patients are more vulnerable to the effects of hypotension as increased vagal tone results in BP drifting to physiologically lower values (150). During this time, collaterals can be inadvertently lost, and for patients admitted with persistent occlusions who are being monitored for collateral support frequent examination and monitoring of BP (preferably with an arterial line to provide rapid, real-time data) is advised.

These concerns are further highlighted in the transfer of patients between institutions. Before 2015, telemedicine was utilized to provide expert neurologic care to stroke patients at centers where no neurologist was available. The role of Telemedicine is evolving with the new stroke trials, with a widely practiced model being the hub-and-spoke model, where a large tertiary care center serves as the “hub” for smaller community “spoke” hospitals (151). Although current Telestroke models have been shown to safely and efficiently aid in administering tPA to eligible patients, a prominent concern has been time delays for the delivery of ET. Transfer delay from spoke hospitals, where ET is unavailable, to the hub hospital was identified as a common reason that acute stroke patients with LVO were excluded from ET in a Chicago-based Telestroke model (152). Implementing interventions that preserve ischemic tissue until recanalization can occur may provide benefit, including minimizing hypotension, improving cerebral perfusion through modification of BP, and using alternative means to promote penumbral sustenance.

## Other Experimental Approaches

Mechanical devices, such as an intra-aortic balloon catheter, have been developed with the idea to augment blood flow. In an animal model, partial aortic occlusion of the abdominal aorta resulted in increased blood volume above the site of occlusion which corresponded to increased cerebral flow (153). Subsequent studies found that titration of the degree of descending aortic occlusion correlated the enhanced blood flow (154). Unfortunately, the largest human trial to date in acute stroke patients up to 14 h from symptom onset did not show any improved benefit (155). It should be noted, however, that these analyses did not include any large randomized-controlled trials that selected for patients with BP- or position-dependent ischemia.

Ischemic pre-, per-, and post-conditioning are other ideas that are being actively explored. Ischemic pre-conditioning involves purposefully inducing brief ischemia to muscle tissue (e.g., a limb with a BP cuff inflated to supra-maximal values and then clamped for a period) to protect against subsequent uncontrolled ischemia in the setting of myocardial infarction or stroke. This idea was first described for cardiac tissue in 1986 (156) and later described in the brain (157). However, in the case of acute stroke, pre-conditioning is not a feasible approach given its unpredictability. This led to animal studies that showed per- and post-conditioning, which utilized induced brief ischemia to muscle during and after stroke, respectively. These show promising results (158, 159), and human studies utilizing interventions as simple as BP cuff inflation by first responder services are underway (160).

In addition to reperfusion and enhanced perfusion, other efforts have been focused on developing therapeutic targets that afford neuroprotection. Interestingly, patients with BP- or position-dependent ischemia clinically or those with imaging demonstrated penumbra serve as an ideal model to test novel agents as there is salvageable tissue. The success of the 2015 ET trials previously described is a testament to the importance of patient inclusion criteria. There is a crucial need to develop pharmacologic agents to preserve penumbra and extend the window for ET (161). In addition, after obtaining reperfusion post-thrombectomy, these agents may have synergistic effects with reperfusion therapies since they can more easily reach their intended target to act on damaged tissue. During acute ischemic stroke, there are numerous mechanisms involved in cell death. Major targets have been neuronal death, white matter changes, and inflammation and microglia (28, 29, 162). Despite this, to date, no agent has proven effective in robust, randomized clinical trials.

Regarding neuroprotective strategies, some of the most common agents studied are NMDA receptor antagonists. These have been developed as they are proposed to reduce the excitotoxicity in acute stroke. The first to be tested in humans was Dextrorphan; however, side effects of hallucinations and hypotension limited its use (163). NA-1, thought to reduce NMDA mediated injury through effects downstream by inhibition of PSD95, was used in the ENACT trial which pretreated patients undergoing endovascular aneurysm repair. This procedure has a high rate of embolic infarcts so it could be utilized as an effective pretreatment model in humans. Interestingly, NA-1 administration was associated with reduced MRI lesions (164). A Phase 3 trial is evaluating this agent currently (FRONTIER) (165), and there is also a trial underway evaluating its use in patients undergoing ET (166).

Magnesium has been explored as another viable option because in addition to blocking the NMDA receptor, it may also increase regional blood flow and block voltage-gated calcium channels (167, 168). Magnesium has been further shown to have an effect on white matter (169). In the intravenous magnesium efficacy in stroke study, magnesium given within 12 h did not affect mortality or disability at 90 days (168). FAST-MAG was a unique trial that administered magnesium in field within 1 h of symptom onset for the majority of patients. This illustrated the feasibility of a delivering a drug to preserve penumbra en route to a facility capable of ET. Unfortunately, there were no improved outcomes with this therapy (170). Calcium channel blockers were evaluated for their potential to reduce excessive calcium influx. Most recently, Nimodipine given within 6 h failed to yield benefit (171). Their role in preventing vasospasm in stroke has yet to be determined though, as efficacy has been shown in subarachnoid aneurysm rupture (172, 173). Treatment with NXY 059, which was designed to trap free radicals and decrease excitotoxicity, showed improved mRS at 90 days in the SAINT I trial (174). *Post hoc* analyses also showed decreased hemorrhages when given with tPA, but these data were not replicable in SAINT II (175, 176).

Several agents were designed to minimize inflammation, edema, and reperfusion injury. Glyburide is a selective inhibitor of the sulfonylurea receptor. It is thought that it may limit edema formation by multiple mechanisms, including perhaps reducing matrix metalloproteinase 9 (MMP-9) activation. This has been suggested in retrospective data in diabetics (177, 178) and has been currently prospectively evaluated after promising early results (179). Other neuroprotective agents acting through MMP-9 include minocycline and edaravone. Minocycline has been evaluated for safety and ability to reduce MMP-9 levels (180, 181). It and other tetracycline antibiotics have been shown to reduce leukocyte infiltration (182). Edaravone, a free radical scavenger, has shown early promise but awaits a large randomized trial (183). In a combined therapy with cilostazol, it was shown to be safe and did improve functional outcome in a small study (184). Enlimomab can block an intracellular adhesion molecule thereby preventing adhesion of inflammatory cells to the vessel wall (185). Unfortunately, patients treated with this drug had an increase in fevers, which may have confounded results. Another potential therapeutic target is a protective axis of the renin-angiotensin system known as the ACE2-Ang-(1-7)-Mas axis (186–190). Early data are examining this system in human patients (191).

In contrast to neuroprotective agents, which act acutely, agents designed to augment neural repair are thought to act primarily from weeks 2 to 12 and beyond in humans. For LVO patients with potential large areas of cortical and white matter injury, these agents may hold great promise. Carmichael reviewed several underlying targets (192), including axonal sprouting, neurogenesis, gliogenesis, and changes in neuronal excitability in peri-infarct tissue. Timing is critical, as many agents that augment repair can have deleterious effects in the acute setting. Perhaps the most widely used agent to date, Fluoxetine, is a selective serotonin reuptake inhibitor (193, 194). The strongest evidence of its efficacy derives from the FLAME trial which examined motor recovery at 90 days (195) and other smaller trials (196–199). Fiblast, a basic fibroblast growth factor, showed initial promise but was terminated in a large study for poor risk-benefit ratio (200). Mesenchymal stem cells have been determined safe, and efficacy studies are ongoing (201). A recent trial showed efficacy in a small group of patients (202). Cerebrolysin, a peptide preparation with pleiotropic effects including multiple neuroprotective and neural repair properties, was shown to improve motor recovery at 90 days in the CARS trial (203). GSK249320, a monoclonal antibody promoting axon outgrowth by inhibiting myelin-associated glycoprotein, has been determined safe, but no efficacy studies are in progress (204).

## Summary and Future Directions

The field of stroke has entered a new era of care with several recent trials demonstrating efficacy and safety of ET for LVO patients. The success of ET in selected patient populations encourages research aimed at expanding the number of patients who can be eligible for this highly effective therapy and focusing efforts on identifying therapeutic approaches that target penumbral sustenance. Preserving penumbral tissue and preventing core growth will increase the number of patients eligible for ET and further improve outcomes for patients undergoing this therapy.

Collateral circulation plays a key role in penumbral preservation. The cerebral vasculature has a unique ability to autoregulate. This is lost in acute stroke, resulting in pressure passivity. Patients must be carefully selected for ET and other therapies based on a mismatch between the area of stroke infarcted and the ischemic area that is hypoperfused but still viable. Although optimal BP targets are still debated, there are likely shifting needs depending on the type of stroke, the acuity of stroke, factors related to ET, and the success of recanalization. Moreover, these BP targets should be patient-specific, taking into account a host of factors that influence risk from hypo or hypertension (permissive or induced).

Effective modulation of BP allows optimization of patient outcomes. Current principles and targets are based on limited published data and pathophysiological understanding, but future directions will be based on randomized trial data and will allow better modulation of these variables in the care of LVO patients. BP manipulation is one component of effective penumbral sustenance, though other possibilities, such as neuroprotection, continue to be explored with promise. The health of the neurovascular unit may be promoted with these agents acutely, and there may be synergism with reperfusion strategies as blood flow is restored to tissue at risk. In addition, agents that enhance neural repair of already damaged tissue will be important as more patients will survive the acute stroke period in this new era. The frontier of this science is dependent upon consistent clinical approaches to the care of LVO patients and either prospective studies or careful registry of patient data so that outcomes can be examined. Interventions with the greatest potential must be prioritized and studied using robust, randomized, placebo-controlled trials.

## Author Contributions

RR, TL-M, and AD conceived the idea, prepared the manuscript, and edited the manuscript. CS, RC, JR, and JH edited the manuscript.

## Conflict of Interest Statement

The authors declare that the research was conducted in the absence of any commercial or financial relationships that could be construed as a potential conflict of interest. The reviewer, NY, and handling editor declared their shared affiliation, and the handling editor states that the process nevertheless met the standards of a fair and objective review.
